# Social Value and Urban Sustainability in Food Markets

**DOI:** 10.3389/fpsyg.2021.689390

**Published:** 2021-06-16

**Authors:** Beatriz Guzmán-Pérez, María Victoria Pérez-Monteverde, Javier Mendoza-Jiménez, Cándido Román-Cervantes

**Affiliations:** ^1^Cátedra Cajasiete de Economía Social y Cooperativa, Universidad de La Laguna, San Cristóbal de La Laguna, Spain; ^2^Departamento de Economía, Contabilidad y Finanzas, Universidad de La Laguna, San Cristóbal de La Laguna, Spain; ^3^Departamento de Dirección de Empresas e Historia Económica, Universidad de La Laguna, San Cristóbal de La Laguna, Spain

**Keywords:** social sustainability, urban food markets, cooperatives, integrated social value, Canary Islands

## Abstract

Urban food markets can promote sustainable development through the generation of social value in the spaces where they are located and contribute to sustainability on a global scale. To measure this, indicators are required to evaluate and monitor these markets. Studies in this regard are scarce and often developed according to top-down schemes. This study seeks to remedy this relative deficiency and aims to design specific social sustainability metrics for these organizations from a bottom-up perspective. The Integrated Social Value model is used. This social accounting system is considered appropriate in this study due to the phenomenological approach on which it is based and is applied to a service cooperative located in the Canary Islands. The main contribution of this work is that new social sustainability indicators are obtained and applied to the analysis of an entity, and they are relevant and understandable to stakeholders. This would provide, in future developments, a system of sustainability indicators for similar organizations in Spain.

## Introduction

The Brundtland Commission's conceptual approach to sustainable development (Brundtland et al., 1987) is interpreted by the United Nations from an integral perspective, gravitating around the person and involving the provision of material well-being and freedom for present and future generations (United Nations Environment Programme, 2005). This institutional vision of development is considered relevant because, currently, it is guiding the efforts of countries and the international community to achieve a more sustainable world by 2030 (United Nations, 2015). To do this, decisive action is required at the local scale, due to the strategic capacities on which the paradigm of the new development model is based (Tomás-Carpi, 2008b).

Social Economy entities have been recognized as key agents in the action plan to achieve sustainability, both at the institutional level (United Nations, 2015) and the academic one (Chaves and Monzón, 2019) for two main reasons. Firstly, the principles of Social Economy entities are aligned with the values that guide sustainable development (Utting, 2015), so they can promote the human and integral development model that is intended (United Nations Inter-Agency Task Force on Social and Solidarity Economy, 2016). Secondly, they carry out their activity essentially at the local level, favoring human, and inclusive development at this territorial level (Mozas and Bernal, 2006).

Among the diversity of spaces, urban ones receive special attention, as they are territories that concentrate approximately two-thirds of the world's population. Thus, the effective scope of a more sustainable planet will depend, to a large extent, on their development models and behavior patterns. The promotion of sustainability in these urban areas is specifically addressed in the United Nations New Urban Agenda (United Nations, 2017). This is an action plan that guides the European Union and its Member States, and in which the Third Sector is explicitly recognized as a strategic vector for the achievement of an inclusive and integrative development.

In the words of the Belgian historian Henri Pirenne (1862-1935) “*cities are the daughters of commerce”* (Casares and Rebollo, 1997, 91), and such commercial exchanges, today, continue to play a relevant role in these spaces. In particular, urban food markets, traditionally owned by the municipality, are reference points for the supply of basic products in cities (Casares, 1999). In addition to a clear orientation toward proximity, which is an element of value in sustainable urban development, they have been recognized for their ability to regulate prices, as well as to revitalize neighborhoods and historic centers (Costa et al., 2015). These are strategic aspects in the New Urban Agenda, to which the positive externalities of rural territories derived from the particular urban food system that urban markets promote could be added (Pothukuchi and Kaufman, 1999; Winter, 2003).

The functions of municipal food markets go beyond the sale of goods for human consumption and their impact on the local economy of proximity. Additionally, there is an intangible impact on the network of immaterial relationships in the social sphere that usually occur around these successors to the ancient Roman forums. It is the relationships between people and the social perceptions of the market that links them to the Social Economy. They are, without doubt, places of exceptional exchange, and sociability (Cantero, 2007).

However, recent trends in privatization, touristification, or exclusion that have occurred in these commercial spaces have come to question these socioeconomic functions and, consequently, their positive effect on sustainability (Crespi-Vallbona and Domínguez-Pérez, 2016). The research that has dealt with analyzing urban food markets is scarce with only that of Crespi-Vallbona et al. (2017) for Spain, applied to the Boquería Market in Barcelona and managed by its merchants themselves under an associative form of the Social Economy. The sustainability metrics proposed in their study are designed following a top-down approach, and the research method is programming by objectives (Schniederjans, 1994).

Our research seeks to reduce this relative scarcity in the literature, establishing as the main objective a system of indicators of social sustainability specific for these markets. This is carried out following a bottom-up approach, and from the perspective of measuring the social value urban food markets generate. The Integrated Social Value model is used (Retolaza et al., 2016), and it is applied to the case study of the *Mercado de Nuestra Señora de África* (Market of Our Lady of Africa), located in the city of Santa Cruz de Tenerife (Canary Islands), and managed by a cooperative company. The main contribution of this research is that it provides a system for monitoring the contribution to the social sustainability of an urban public space managed under a Social Economy framework. Therefore, it reinforces the integration of its agents within the Spanish Urban Agenda (Ministerio de Fomento, 2019).

## Theoretical Framework

### Urban Sustainable Development and the Social Economy

Sustainable development has become the accepted cornerstone of most political and social agendas since the oft-cited Brundtland Commission established the first universally accepted definition of the concept. This acceptance has not been without criticism, which is usually based on the abstractness of the concept. The vagueness and indeterminacy present in it (Daly, 1996) has given rise to multiple interpretations and, consequently, to diametrically opposed proposals to achieve sustainable development. It has sparked intense discussions, at least, around two questions. First, what needs are there to be covered or satisfied, only material ones, related to the standard of living and the consumption of basic goods and services (Solow, 1974), or if there are also ones concerning the expansion of decision-making capacity (Anand and Sen, 2000). Second, what type of capital conditions growth (United Nations Development Programme, 2011), is it technological capital (Solow, 1995) or natural capital (Barbier et al., 1990; Ross, 2009).

Beyond the conceptual debate, recent years have seen the consolidation of cities as a central element to achieve a sustainable development model. According to data from the World Bank, 55.74% of the population lived in cities in 2019 (The World Bank, 2021) and the United Nations forecasts that this number will reach 70% by 2050 (United Nations Department of Economic Social Affairs Population Division, 2019). This massive displacement from rural to urban areas puts more pressure on effective city planning of these environments. Sustainable urban development has been defined as “a result of the drive for cities to be more responsive to citizens' needs, offer conditions that promote high quality of life and sustain and enhance competitiveness in an increasingly globalized environment” (Angelidou et al., 2018, 1).

Urban development policies affect key components of the environmental dimension of sustainable development, such as GHG emissions (Pan et al., 2020), water availability and its management (Mikovits et al., 2018). They can also shape social aspects like partnership relationships between different stakeholders (Xue et al., 2020) and even citizens' engagement in decision-making processes (Falco and Kleinhans, 2018).

The importance of sustainable urban development in the global agenda is confirmed by the Sustainable Development Goals (SDGs) established by the United Nations in 2017, which specifically included one devoted to this goal. Goal 11 can be found under the heading: *Make cities and human settlements inclusive, safe, resilient, and sustainable*. This goal includes a series of targets that address key issues that have been studied in the field of urban development such as affordable housing (Immergluck and Balan, 2018), cultural heritage (Guzmán et al., 2017), or waste management (Serge Kubanza and Simatele, 2020).

Social inclusion is also mentioned among the targets of the United Nations. Social aspects are gaining more importance as key components of sustainable urban development, especially those related to the transformation of traditionally deprived areas (KriŽnik, 2018). In this context, the Social Economy has been considered a necessary partner to enrich governance processes leading to sustainable cities (Kaufmann and Sidney, 2020). Despite being recognized as a key contributor to sustainable development at both institutional (United Nations, 2015, 2017) and academic levels (Falcón and Fuentes, 2017; Chaves and Monzón, 2019), it still plays a minor role in the definition of urban policies. However, starting from the definition of Social Economy entities as being “organizations with explicit social or environmental objectives guided by principles and practices of cooperation, solidarity, ethics, and democratic self-management” (United Nations Inter-Institutional Working Group on Social and Solidarity Economy, 2014), there are several arguments that support a greater implication of the Social Economy in this area.

First, its operating principles are aligned with the values that guide action toward a more sustainable planet, so they promote the human and integral development (UNTFSSE, 2016). In this regard, the defining features of Third Sector entities could expand the freedoms of present and future generations (Monzón Campos, 2013). Such principles and values would affect social utility (Gadrey, 2005) and sustainable development (Connelly et al., 2011; Chaves and Monzón, 2012; Rahdari et al., 2016; Kim and Lim, 2017). Moreover, the social purpose or democratic governance that characterizes it would allow organizations to exercise social, economic, and political functions in an economic system that would contribute to sustainable and socially inclusive growth (Chaves and Monzón, 2012; Mozas, 2019).

Second, the production, finance and exchange patterns of Social Economy entities prioritize not only people, but also respect for the natural environment. Hence, it is estimated that its action would holistically and synergistically reinforce the different dimensions of sustainability, constituting an alternative to the unbalanced and exclusive development based economic model (Bono, 2012).

A third reason why these entities are considered critical for meeting the sustainable development challenge is related to the space in which they operate. In this sense, their roots in the local economy and the community in which they operate would favor sustainable development at this territorial level (Mozas Moral and Bernal Jurado, 2006; Poyatos and Gámez, 2009; Connelly et al., 2011). Consideration of local demands and needs and achieving them in practice is of the utmost importance in effective people-centered development. These characteristics can be useful to tackle one of the problems encountered during the application of the Millennium Development Goals, predecessors to the current SDGs, which was their global and national definition, thus ignoring local demands and, therefore, the level of achievement of the goals at this level.

Lastly, the potential of the Social Economy for deployment toward the SDGs can also be explained on the basis that it acts not only through the entities that constitute it, but also through the civil society, which has been urged to collaborate to achieve these objectives (Chaves and Monzón, 2018; Mozas, 2019).

### Socio-Economic Functions of Food Markets in the Twenty-First Century

Commercial exchanges have been one of the main driving forces of the structural and economic development of Western European cities, even since the classical civilizations of the Mediterranean (Cameron, 1992, 56–68). Public spaces or squares in which markets took place have evolved to this day. Current urban food markets are a modern expression of the ancient Greek agoras or Roman forums (Casares and Rebollo, 1997, 91). In Spain, they are conceived as “a set of retail establishments, grouped in a building, which have a common operation management controlled by a city council or another entity by concession of the latter” (Casares and Rebollo, 1997, 91).

From the different “families” inside the Social Economy, food markets that adopt a cooperative structure are probably the ones that have been most related to urban development due to their characteristics and influence on the environment. The original function of food markets was the supply of fresh products of basic foodstuffs to the urban population, with guarantees of quality and at relatively favorable prices. This has been maintained until today, although other forms of commercial distribution have also taken on this role. This supply value of basic goods by the markets manages to project itself into the future (Rebollo and Casares, 2006, 26) thanks to a series of social and economic aspects (Casares, 1999). Specifically, the promotion of agglomeration economies and the consequent facilitation of purchasing by users, the zonal regulation of prices and competition, or the production of services such as the storage and preparation of products. These aspects have allowed the “iron markets” from the nineteenth century to respond to the needs of customers of the twenty-first century, adapting to the new conditions of demand.

Moreover, the competitive orientation regarding the proximity that characterizes food markets (Casares, 2003) has a positive impact on the quality of life of their users, as well as being beneficial for the cities in which they are located. In this sense, they promote a particular urban food system (Pothukuchi and Kaufman, 1999; Winter, 2003; Costa et al., 2015) characterized by enabling regional rural development and co-urbanization (Casares, 2003), contributing to preserving the environment and local uses and customs, as they are often settings for local gastronomy and autochthonous social relationships (Crespi-Vallbona and Domínguez-Pérez, 2016, 4; Alsadaty et al., 2020). Indeed, they are public spaces for socialization, intercultural exchange (Crespi-Vallbona and Dimitrovski, 2017) that can favor social integration and inclusion (Hiebert et al., 2015).

The proper functioning of these markets also generates positive externalities on adjacent areas, promoting outsourcing and economic dynamization (Casares, 1999, 26), revitalizing neighborhoods and historic centers (Martín-Rojas, 1999; Costa et al., 2015; Dimitrovski and Crespi-Vallbona, 2017). Despite their relative importance, traditionally, only internal aspects have been analyzed, such as the profile of merchants (Arocutipa et al., 2018), workers' conditions (Pita, 2019), or sanitary conditions (Calizaya Limaco et al., 2010).

Moreover, in recent years, there has been a growing interest in the role of these entities as tourist attractions in cities. According to Henche (2017) traditional food markets are becoming a cultural attraction, specially related to gastronomy and local life-style promotion that can help to promote minoritarian types of tourism, like gastrotourism and experiential tourism, that can be an alternative to the exhausted and predominant mass tourism. Fusté Forné et al. (2020) highlight that fresh food is an attractor for both locals and tourists, who can appreciate the authenticity and particular features of the area. Therefore, urban food markets are places appreciated by those who travel for cultural and gastronomic reasons (Crespi-Vallbona and Domínguez-Pérez, 2016; Crespi-Vallbona and Dimitrovski, 2017). This aspect can lead, however, to the implementation of initiatives aimed at attracting and encouraging the visit of travelers and tourists, or touristification. Those initiatives, instead of promoting the conservation of local uses and customs, could consist of offering gourmet products, or holding events that are not a reflection of local culture. This attempt to convert urban food markets into focal points of tourist attraction is sometimes accompanied by the renovation of the surrounding spaces and neighborhoods. A spatial regeneration that would lead to higher prices for shops and homes and, ultimately, to gentrification, or replacement of traditional neighbors, generally belonging to the popular classes, by others from more affluent classes (Crespi-Vallbona and Domínguez-Pérez, 2016). Both processes, touristification and gentrification, therefore, promote dynamics that would counteract some of the beneficial effects previously described (Crespi-Vallbona et al., 2017).

### Measuring Social Sustainability in Social Economy

Although the development of indicators for measuring sustainability has constantly evolved in the last 25 years, research carried out on their social aspect seems to be at a more incipient stage of development than the economic and environmental dimensions. The measurement of the social dimension of sustainability is particularly concerned with the organization's impacts on the social systems in which it operates, as well as the organization's relationship with its various stakeholders (Labuschagne et al., 2005). For the indicators of organizational social sustainability to be relevant, that is, “measure the phenomena intended to be measured” (Miller, 2001, 352), they must refer to the activities or outputs of the organization and linked to outcomes or social impacts on the environment (Milman and Short, 2008; Searcy, 2012).

Obtaining relevant social sustainability metrics involves a certain complexity, mainly due to the fact that many of the social outcomes and impacts are outside of corporate control, are difficult to characterize and, with some frequency, are based on value judgments (Keeble et al., 2003). Hence, on many occasions, the selection of the indicator is guided more by what can be measured than by what should be measured (Milman and Short, 2008). The indicator therefore ceases to fulfill its fundamental objective, linking cause and effect, which is problematic, as it does not guide decision-making toward the end pursued (Milman and Short, 2008).

For measuring the social impact of organizations, dialogue with stakeholders and understanding what is relevant to them is considered a particularly appropriate approach (Costa and Menichini, 2013; Costa and Pesci, 2016). Likewise, an in-depth understanding of stakeholder values and interests through a phenomenological perspective is recommended (Deery et al., 2012).

Among the proposals to determine the social impact at an organizational level the following can be highlighted: Balance of the Common Good (Felber, 2012), B Impact Assessment (Honeyman, 2014), Capacity Index (Garriga, 2014), Analysis of the Integrated Social Value (Retolaza et al., 2014), or the Social Return on Investment. The metrics that these systems propose refer to different links in the impact value chain, as well as requiring different degrees of participation from the entity's interest groups (Ayuso, 2018).

A breakthrough that has facilitated impact assessment for organizations and guides their activities toward the SDGs is the alliance between the United Nations Global Compact, Global Reporting Initiative (GRI), and the World Business Council for Sustainable Development (WBCSD) which has established an inventory of indicators allowing entities to directly select those that are considered most relevant to them or to use it as a source of inspiration to define their own indicators (UN GLOBAL COMPACT, GRI, and WBCSD, 2015). This inventory links the SDGs and their targets with a series of business themes to which it then adds indicators extracted from different sources. This first (and laudable) effort to determine a more extended system for measuring sustainability is, however, incomplete, and needs further development in the future both to complete all aspects and to adapt it to different sectors.

In the specific case of the Social Economy, the Interdisciplinary Group of the United Nations in Social and Solidarity Economy (UNTFSSE) and the Group of Experts Social Economy and Social Enterprises of the European Union (GECES) have, among their purposes, to make visible the contribution of the entities that integrate this sector to sustainability (UNTFSSE, 2016, 2017). This task is being tackled not only by adapting the existing frameworks at different aggregation scales to the reality of the sector, but also by developing, in parallel, specific models that reflect the peculiarities of the agents that comprise it.

From the point of view of generic systems, sector accounting has been applied to the Third Sector (Barea and Monzón, 2006). This framework measures the contribution of the Social Economy to economic sustainability. At the academic level, the impact in terms of employment or contribution to GDP of certain branches of productive activity or families of the Social Economy, mostly cooperatives, has also been addressed (Foncea and Servós, 2010; Mozas, 2019).

At an organizational analytical level, Social Economy entities have used systems to quantify their social impact such as the Integrated Social Value (Retolaza et al., 2014, 2015; Etxezarreta et al., 2018; Lazkano et al., 2019; Ayuso et al., 2020a; Guzmán-Pérez et al., 2020; Román-Cervantes et al., 2020; Ruiz-Roqueñi, 2020), or the Social Return on Investment (Rotheroe and Richards, 2007; Ariza et al., 2018). At the organizational level, Bassi and Vincenti (2015) have also developed the Social Added Value Evaluation, S.A.V.E, applying it to three Italian social cooperatives. Monzón Campos (2013) also develop a table of key performance indicators based on key areas, consistent with the characteristics of social enterprises. Bagnoli and Megali (2011) and Arena et al. (2015) propose specific performance measurement systems for social enterprises, applying them to Italian entities. Moreover, Bull (2007) adapts a balanced scorecard (Kaplan and Norton, 1996) to the reality of social enterprises, applying it to a sample of 30 companies in the United Kingdom.

Within this general framework, studies that have dealt with measuring the sustainability of the urban food markets are scarce. Casares and Rebollo (1997) offer an approximation of the economic dimension of the sustainability of urban food markets in Spain, providing data on commercialization volumes, market share, or sources of supply. Crespi-Vallbona et al. (2017) propose the analysis of sustainability for the Boquería Market (Barcelona) using programming by objectives (Schniederjans, 1994) by designing a system of 42 indicators, based on the Sustainable Tourism Attitude Scale (Choi and Sirakaya, 2006), European Commission (2013) and the academic literature on measuring the sustainability of tourism. Finally, outside of Spain, Alsadaty et al. (2020) point out the qualitative factors that, from the point of view of sustainable development, must be considered in the socio-spatial regeneration of the historical Attaba market in Cairo.

There is a certain lack of studies that propose social sustainability metrics for urban food markets in which dialogue with stakeholders is used. This study tries to answer this research gap using the generic ISV model. This system has been chosen mainly for the following two reasons. In the first place, because for the measurement of social value a dialogue is opened with the stakeholders to understand in depth what their interests are, through the phenomenological perspective (Ayuso et al., 2020b). Secondly, because the indicators that are raised refer to the organization's outputs linked to such interests. Additionally, with this system the indicators or outputs are re-expressed in monetary units, a unit of measurement that has the advantage of facilitating the understanding of the information provided to various stakeholders for their decision-making.

## The Integrated Social Value Model

The ISV model (Retolaza et al., 2014, 2016) is a social accounting system, or “accounting that goes beyond economic-financial aspects” (Gray, 2002). It is characterized by combining qualitative and quantitative analysis to express in a single monetary measure the value that an organization generates, and its stakeholders perceive as such (Ayuso et al., 2020b).

This model is based on the perspective of stakeholder theory (Freeman, 1984; Freeman et al., 2010; Retolaza and San-Jose, 2011), and it conceives the value created by an organization in a broad way. It considers both the value generated to the owners of the capital and the value distributed to the stakeholders, as well as the value traded in the market, including the value transferred outside the price system (Freeman et al., 2020). This broad value, called ISV can be considered similar to blended value (Emerson et al., 2003) or shared value (Porter and Kramer, 2011).

The ISV model starts from the premise that an entity's results are transformed into value only when someone else values them (Retolaza et al., 2014, 2016). This analytical method is the phenomenological approach (Moustakas, 1994), which implies understanding the meaning for people who are related to or are affected by an entity, which implies the experience of interaction with it or what results from it. This perspective involves approaching social value through stakeholders' perceptions, without having previously established the dimensions in which this value is materialized (Retolaza et al., 2016). These perceptions of social value are considered from the perspective of fuzzy logic (Zadeh, 1965; Kaufmann and Gil-Aluja, 1986), which makes it possible to quantify in monetary terms the social value created by the organization and perceived by its stakeholders.

**Figure 1:**
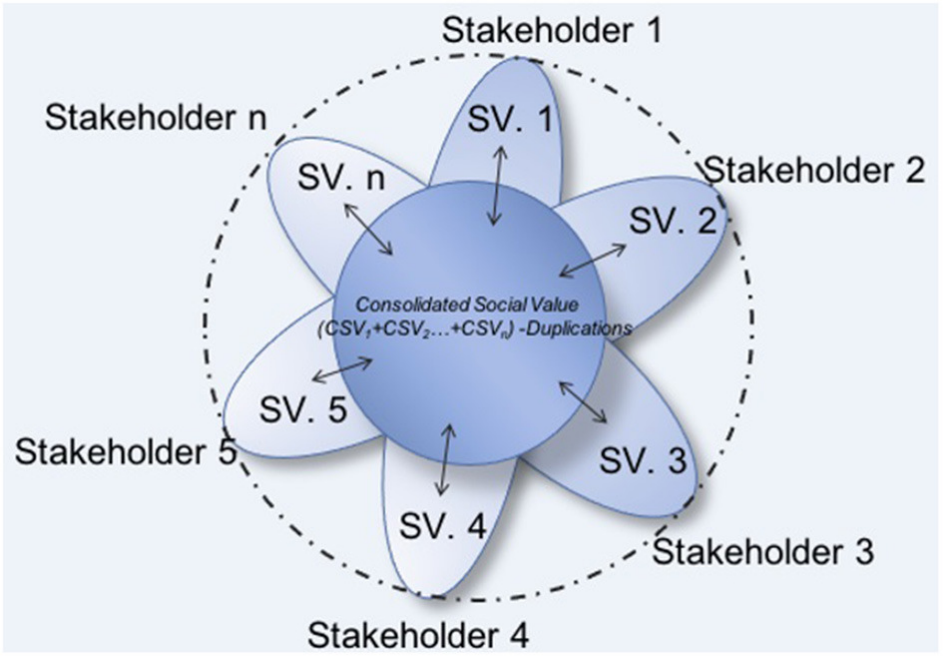
Graphical representation of the ISV model. Source: adapted from Retolaza and San-Jose (2016).

ISV according to this system is not unique or common for all stakeholders, as their perceptions are different. The graphic representation of ISV is shown in Figure 1 and can be considered as being like a flower. The area of the inner circle reflects the social value shared by the stakeholders, and the area of each petal reflects the value generated by the entity and perceived by a particular group, without converging with the rest.

To objectify and quantify the ISV in monetary terms, a standardized process is followed consisting of the following four stages: (1) identification of interest groups; (2) identification of value variables; (3) monetization of the indicators; and (4) calculation and visualization of the ISV (Retolaza et al., 2016). Qualitative analysis is used in the first two phases, and quantitative analysis in the last two (Ayuso et al., 2020b).

The objective of the first stage is to identify the stakeholders for whom the entity creates value. Although there are different criteria to categorize and prioritize interest groups (Savage et al., 1991; Clarkson, 1995; Mitchell et al., 1997), the ISV system seeks to specify the groups that receive some type of created value by the entity, whether or not it is monetary (Freeman et al., 2020). In the second stage, the aim is to identify the variables or dimensions of value perceived by stakeholders. This identification is carried out through a dialogue with stakeholders in which they openly express the value they perceive from the entity, using in-depth interviews as the data collection technique. The analytical structuring of the qualitative information collected is carried out following the process of the phenomenological approach: the set of perceived values is grouped into thematic areas, the value variable in the different areas is identified, and the content of the value variables is described in technical or scientific language (Giorgi and Giorgi, 2003).

In the third stage of the ISV analysis, the dimensions of perceived social value are quantified in monetary units, identifying measurable indicators or outputs of the organization linked to the value variables, and monetizing them according to the accounting criteria of fair value. The process ends with the consolidation and visualization of the different ecosystems of the ISV.

The research method in the ISV model is action research; an approach that seeks to solve a problem while creating knowledge or theory from action (Lewin, 1946). One of its distinctive elements is that the professionals under study actively participate in research. Therefore, the AR team is made up of the researchers themselves and professionals, who interact continuously throughout the entire process (Coughlan and Coghlan, 2002). In the particular case of the ISV model, the research is carried out in collaboration with the stakeholders that are affected.

ISV is a system for measuring social value that is considered especially appropriate for this kind of study, given its main objectives. Firstly, because it is based on the phenomenological approach, a qualitative research method of outstanding convenience for determining social impact (Deery et al., 2012). Secondly, because it allows the design of relevant corporate social sustainability indicators (Ayuso et al., 2020b), as the quantification of social value through fuzzy logic is carried out through the search for measurable outputs of the organization and is linked to outcomes or impacts. Finally, because the monetary quantification of the indicators is understandable for stakeholders, as well as being useful and relevant for business decision-makers (Aguado et al., 2015; Freeman et al., 2020).

This system has been empirically applied to more than 80 organizations of different sizes and industries, both commercial and from the Social Economy (Lazkano et al., 2020). In this last area, it has been used to quantify the social value generated by diverse types of organizations, such as cooperatives (Etxezarreta et al., 2018; Lazkano et al., 2019; Ruiz-Roqueñi, 2020), foundations (Retolaza et al., 2015), non-profit organizations (Retolaza and San-Jose, 2016), Agrarian Transformation Societies (Román-Cervantes et al., 2020), fishers' associations (Guzmán-Pérez et al., 2020) and Special Centers of Employment (Ayuso et al., 2020a).

The model is still in the development stage, and there are some methodological issues that lack standardization (Ayuso et al., 2020b; Freeman et al., 2020). Progress in standardization will facilitate comparability between similar organizations, as well as reducing the discretion and subjectivity present in the attribution of outputs and proxies (Ayuso et al., 2020b).

## Analysis of the Social Integrated Value of the Food Market Nuestra SeñOra de áfrica

### Case Study

The Mercado Nuestra Señora de África (MNSA), popularly known as La Recova, is one of the food markets of the Canary Islands, located in the city of Santa Cruz de Tenerife (Canary Islands). It is the successor to the Old Recova of 1852. It was built by General Ricardo Serrador Santés of the headquarters of the Economic Command in the post-war period and inaugurated in early 1944. This Market takes its name from the Virgin of Africa, Patron Saint of Ceuta.

The aesthetic design of the emblematic building in which this municipal market is located presents a neoclassical colonial air with a modern architectural and urban sense. Its main features are a large arch, 8 m in radius, a central patio, which resembles a classic Spanish-style plaza, and a Mudejar-style tower. It was declared an Asset of Cultural Interest in 2004.

In its beginnings, it was an important food sales space, both in the city itself and throughout the island, in which wholesale marketing of such goods was also conducted. This wholesale business was transferred in 1974 to Mercatenerife, created specifically for this distribution channel in Santa Cruz de Tenerife. This inevitably led to a reduction in MNSA's wholesale business. However, retail sales were maintained there, although in 1985 many of its retail stalls began to be abandoned as a result of changes in customer purchasing trends (Casares and Rebollo, 1997).

In 1992, the MNSA space, under municipal ownership, began to self-manage through a Cooperative formed by La Recova merchants, which looks after the interests of its members and studies new ways of making the space profitable. This associationism formula continues today, comprising 149 partners. In 2018, it was recognized with awards such as the Gold medal of the Government of the Canary Islands and the Gold medal of the city of Santa Cruz de Tenerife. In 2019, it was recognized internationally by the British newspaper The Guardian, placing it among the top ten in the world, along with the Fez market in Morocco, the Mahane Yehuda market in Jerusalem or the Lau Pa Sat market in Singapore.

Today, it combines an offer of fresh food products, such as fruits, vegetables, meat, and fish, with preserves, charcuterie, and salted fish. It has an attached shopping area where it is possible to buy handicraft goods, books, and bazaar objects. In addition to shopping, it is possible to enjoy Canary Island gastronomy or attend conferences or events such as “The nights of La Recova” (recreational and tasting activities) and “Los sabores de La Recova” (a cooking show) there.

This urban market could be classified as a food market that is a tourist attraction. This type of market is considered of balanced sustainable social use, since it serves the traditional local population as well as tourists. The urban food market offers authentic experiences, typical of the local society and is visited by these new audiences. In this sense, these urban food markets are one of the promoted resources in which locals and tourists can go hand in hand, at least theoretically (Crespi-Vallbona and Domínguez-Pérez, 2016). Therefore, the MNSA promotes sustainable development in the space in which it is located and contributes to the achievement of comprehensive sustainability on a wider scale.

At the beginning of 2019, the management team of the MNSA Cooperative wanted to assess the socioeconomic impact the market was generating on society and go beyond its traditional accounting figures. The fundamental motivation of the organization to carry out the study was to conduct an exercise in transparency and accountability to citizens. In accordance with the participatory research approach of the project, a mixed work team (Action Research) was formed in October 2019, made up of academic researchers and members of the management team of the MNSA Cooperative. The creation of this AR team marks the beginning of the monetization process of the social value of the Cooperative, a process which culminates in December 2020. The sanitary context in which it takes place is that of the COVID-19 pandemic.

### Process of Analysis of the Integrated Social Value

This section presents the results obtained in the different stages of the standardized process that, in the ISV system, must be followed for the monetary quantification of the social value of the entity under study. The metrics established will enable the evaluation and monitoring of the contribution of this entity to social sustainability.

The analysis of the organization's internal documents and the meetings with its management team have made it possible to delimit and identify the interest groups of the Cooperative on a map. After an initial documentary review, groups such as partners, suppliers, customers, workers, or the city council were identified. Subsequent discussions made it possible to expand the initial list of interest groups, and neighborhood organizations, institutions, sponsors, and other markets were also considered. Table 1 contains a synthetic description of the interest groups and the type of relationship that the MNSA Cooperative maintains with each of them.

**Table 1:** Stakeholders description and relationship with the Cooperative of the Market Nuestra Señora de África.

**Stakeholder**	**Description**
Partners	Owners of the commercial stalls located in the Market managed by the Cooperative
Personnel	Staff of the Cooperative that performs the functions of management, administration, counseling, janitorial, maintenance, and cleaning
Customers	They send suggestions for improvement, online opinions, and complaints to the Cooperative
Suppliers	Entities that provide telecommunications services, promotion and advertising, maintenance, and cleaning material
Sponsors–collaborators	Entities that sponsor events organized by the Cooperative
Other markets	Regional and national market federations with which the Cooperative shares experiences, technical, and legal information
City council	Assigns by administrative concession the provision of a municipal public service
Local organizations	Social economy entities, associations and neighborhood entities that the Cooperative supports
Institutions	They provide grants or develop projects in which the Cooperative collaborates

*Source: Prepared by authors*.

To identify the variables of perceived value, the representatives of the MNSA Cooperative and members of the mixed team prepared a list of people considered representative of the different interest groups. The analysis of the interests of the different stakeholders was carried out through 27 in-depth, personal, and semi-structured interviews with representatives of the different stakeholder groups, according to the list proposed by the management team of the Cooperative. Table 2 shows a description of the interviews carried out.

**Table 2:** Interviews with representatives of the Cooperative's stakeholders.

**Stakeholder**	**Description**
Partners	7 holders of positions of diverse commercial activities, with different seniority as partners, gender, and cultural diversity
Personnel	5 representatives: 2 administrators, 2 janitors, and 1 cleaning person
Suppliers	6 representatives of entities that provide different services (IT, digital development, maintenance of electrical equipment, maintenance of water equipment, fire safety, and maintenance of stairs and elevators)
Sponsors–collaborators	1 representative of the sponsoring entity of the event “Las Noches de la Recova”
Other markets	1 representative of the Federation of Supply Markets of the Canary Islands (president of an insular supply market)
City council	1 representative (Mayor of the city)
Local organizations	3 representatives of Third Sector entities, 1 representative of a religious entity in the neighborhood, 1 representative of a university cultural organization
Institutions	1 representative of the institution with the highest frequency of relationship

*Source: Prepared by authors*.

The representatives of the customer group could not be interviewed due to the difficulties to access them, related to the health context in which the field work was carried out (the COVID-19 pandemic). According to estimates by the Cooperative, the number of visitors in 2019 has been 1,060,000 people, with the months from October to March being the ones with the highest influx (120,000 monthly customers on average) and the month of August, the least crowded (40,000 visitors). It is estimated that of this total of clients, 7% are tourists^1^ (73,770 people), and the remaining 93% are local consumers.

The purpose of these interviews is to understand, for the people who are related to or are affected by the Cooperative, what the experience of interaction with the entity involves and the results from it, for the organization they represent or for them individually. The script or protocol for the interview from Retolaza et al. (2016) was adapted to the reality of the Cooperative and its stakeholders.

Interviews were conducted between July and November 2020. Most of them could be carried out in person (18 interviews), although some had to be carried out by telephone (9 interviews), as a preventive measure due to COVID-19 infections. The telephone channel was used to interview 100% of the representatives of the municipal groups and institutions, 75% of the interlocutors of local entities and 80% of those of the supplier group. The average duration of the telephone interviews was 14.2 and 19 min for the face-to-face interviews. In relation to the latter, differences were observed based on the structural characteristics of the spaces in which they were developed: 28.4 min those that took place in open spaces or large rooms (11 interviews) compared to 9.5 min those carried out in offices of smaller dimensions. The groups interviewed in this type of space, closed and small, were staff (60% of their representatives) and partners (43%).

The verbal information collected was recorded in the form of annotations, and then was transcribed. To ensure robustness in this qualitative research phase (Steinke, 2004), the information collected in the interviews was triangulated with the internal documents of the Cooperative and with the information obtained by the academic members of the AR team through direct observation.

Following the recommendations of the literature on qualitative methods (Gioia et al., 2013), the authors independently analyzed the interviews, identifying the thematic areas related to the different dimensions of social value perceived by stakeholders. Initially, 26 first-order variables were identified, which were designated using terms close to those used by the interviewees themselves, such as “one of the best in the world,” “maintenance of the autochthonous,” or “shopping center.” Next, this list of first-order variables was discussed jointly by the academic researchers of the mixed team and reformulated into a consensual list of second-order value variables. For this, technical or scientific language was used that would allow, in the next stage, to link it with measurable outputs generated by the entity and on which it was considered possible to obtain external proxies, such as “collaboration,” “way of life” or “support services. All steps in the data collection and analysis have been documented and can be requested by interested researchers. Table 3 shows the identified value variables and their description.

**Table 3:** Description of the value variables.

**Value variable**	**Description**
Customer attraction	Helps and facilitates suppliers to attract new customers and diversify products
Supporting services	Services provided by the Cooperative, such as legal advice or security, appreciated by the members
Way of life	Allows partners with fewer socioeconomic opportunities to have a livelihood
Social relationships	It favors the emergence of social relations between workers and members of the local community. It encourages the creation of sectoral networks.
Facilitates healthy shopping	It simplifies having the daily purchase of fresh products in the fridge.
Work satisfaction	Reception, labor flexibility, job recognition
Historical heritage	Maintenance of the traditional, the autochthonous. It is part of the history of the city, it is an emblematic place that has a strategic location. It is a tourist attraction
Leadership	Close, cordial, understanding and trustworthy relationship of the management team. Excellence in management
Collaboration	Assignment of spaces, favors the collection of food, predisposition to collaborate
Local consumption	It favors the consumption of regional food products

*Source: Prepared by authors*.

To objectify the values subjectively perceived by the stakeholders, the indicators or outputs of the MNSA Cooperative linked to them have been identified through an intersubjective reflection process. This process has been co-participated by the researchers of the AR team, making decisions on the transformation of variables in a consensual way. For the selection of proxies, or external items that allow an approximation to the monetary value that the outputs could have, the similarity and consideration of the social and temporal context in which the outputs are generated has been taken into account. Since in the ISV model, the choice of proxies is made by applying the fair value criterion (Retolaza and San-Jose, 2018), the market price of an identical output, or one considered similar by the researchers, has been taken as an approximation. The outputs and proxies proposed to monetize the value variables are shown in Table 4.

**Table 4:** Indicators and proxies of value variables.

**Value variable**	**Indicator**	**Proxy**	**Source of the proxy**
Customer attraction	Number of suppliers and collaborators who value it	Price of a sponsored ad on Facebook 5 days, to reach an audience of 15,000 people	Facebook
Supporting services	Number of partners who value it	Difference between the annual cost of advisory, cleaning and security services, and the annual membership fee to the Cooperative	Consultation of prices of companies of advisory services, cleaning and security of the municipality. Cooperative
Way of life	Number of partners with fewer socioeconomic opportunities	Difference between the median annual salary in the province and the Minimum Living Income	Instituto Nacional de Estadística. Salary Structure Survey (Canary Islands). Royal Decree-Law 20/2020, of May 29, which establishes the Minimum Living Income
Social relationships	Number of partners and workers who value it	Annual fee for a social club in the municipality	Consultation of prices of social clubs of the municipality
	Number of groups that value it	Annual fee for a national professional association	Price consultation of regional and national professional associations
Facilitates healthy shopping	Number of partners and workers who value it	Annual amount of weekly home transport of a box of fresh fruit and vegetables	Consultation of prices of island companies that deliver fresh, organic fruit and vegetables
Work satisfaction	Worker surplus	–	Workers
Historical heritage	Amount of competitive municipal grant collection	–	Subsidies for the maintenance, restoration and/or conservation of properties of cultural value during the 2020 financial year—Cabildo de Tenerife. Official Gazette of the Province of Santa Cruz de Tenerife no. 87, Monday 20 July 2020
	Number of tourist visits	Entrance fee to the municipal museum	Price consultation of the Museum Tenerife Espacio de las Artes (TEA)
	Competitive national grant collection amount	–	Aid for projects for the conservation, protection, and dissemination of properties declared World Heritage. 2019. Aid granted to dissemination projects. Resolution 06/06/2019. Undersecretary Ministry of Culture and Sports
Leadership	Producer surplus	–	Suppliers
	Number of individuals who value it	Cost of certification in ISO 9001	Price consultation of ISO 9001 certified companies
Collaboration	Amount of donated resources	–	Cooperative
	Amount of donated resources	Commercial credit interest rate	Maximum APR of the ICO Commercial Credit line effective from 11/30/2020 to 12/06/2020
Local consumption	Savings in the carbon footprint of transporting fruit and vegetables purchased by customers	Social cost of carbon	García-Pérez (2019)

*Source: Prepared by authors*.

For example, for the outputs of the value variables “support services” and “collaboration,” there are identical outputs exchanged in the market, while those of “customer attraction” and “social relations” have been equated to a sponsored ad on Facebook and the membership of a social club, respectively. The criteria followed for the selection of proxies have been those of the ISV model (Retolaza et al., 2016): the costs or savings for the Administration, the territorial unit in which the MNSA Cooperative is located, and the specific period for which data is reported, 2019.

For example, for “aid for the conservation of buildings of cultural value,” “the subsidies granted by the Cabildo de Tenerife” have been taken as a proxy, and for “price of home transport of a box of fruit,” that of companies that provide the same service, located on the island of Tenerife. Regarding choices of proxy values, in the cases in which the variability of said value was high, the centroid score has been taken, in order to optimize the centrality of the membership function (Retolaza et al., 2016). This has been the criterion followed for the choice of the value of “support services” or “social relations.” Finally, for the monetary determination of the value variables, the relational algorithms between outputs and proxies are identified (Retolaza et al., 2016), adopting a form of multiplication in most cases.

### Results of the Analysis of the Integrated Social Value

The information collected in the first three stages of the ISV analysis process and the Cooperative's accounting data allow to calculate the two ecosystems of social value that conform the ISV: (1) the Socio-Economic Value (SEV), or social welfare generated with the performance of economic-productive activity, directly and indirectly (through purchases from local suppliers); and (2) the Specific Social Value (SSV), or non-market social value distributed among the stakeholders, obtained by adding the monetary value of the previously identified value variables.

The SEV is the value generated and distributed by the MNSA Cooperative to its stakeholders, derived from the provision of services to members and transferred in the market in exchange for a price. Salaries paid to workers, taxes to the Public Administration, interest on financial loans, amortizations, and operating income are part of the SEV. These values can be extracted from the financial accounting of the entity. Likewise, the added value mobilized by the Cooperative through purchases from local suppliers is considered within the SEV. To quantify this mobilized value, the structure of the value distribution of local suppliers is analyzed, consulting their commercial data in the Sistema de Análisis de Balances Ibéricos and obtaining the ratios in relation to the annual turnover (Retolaza et al., 2015).

**Figure 2:**
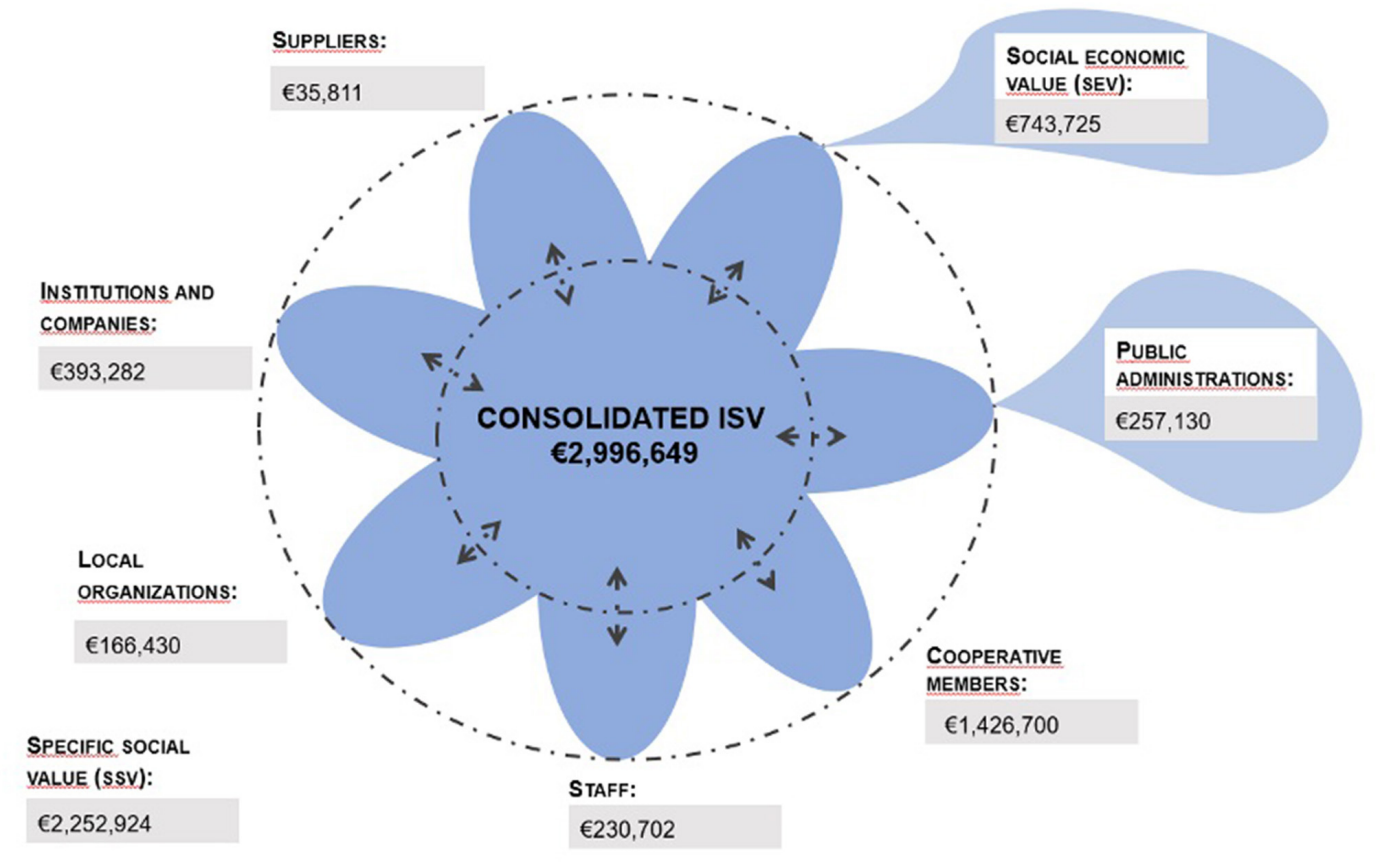
Distribution of the consolidated ISV by stakeholders. Source: Authors' elaboration.

The SSV is obtained by adding the monetary value of the set of value variables. Figure 2 graphically represents the results for the Cooperative of both ecosystems of social value, as well as the consolidated ISV. This consolidated ISV is similar to the equivalent accounting concept, and represents the value created after eliminating duplicates of shared value, created simultaneously by several stakeholders or several types of value.

The consolidated ISV created by the MNSA Cooperative in 2019 is € 2,996,649. The non-market value or SSV for that year (€ 2,252,924) far exceeds the value resulting from the economic activity of the SEV entity (€ 743,725). The amount of SEV that is distributed to Public Administrations is € 257,130. However, taking into account that the Cooperative manages a municipally owned market through an administrative concession, the social value to the Administrations would include, in addition to the SEV, the SSV that the entity generates through its management. This value is included in the variables “leadership” (€ 106,972), “equity” (€ 542,491), “social relations” (€ 76,118), and “local consumption” (€ 69,192). These variables are not exclusive to the City Council and its agencies, but are shared by partners, suppliers, workers and sponsors. This indicates that, in the event that the Cooperative would not assume the management of the MNSA, an alternative directive and management structure would be necessary that is capable of maintaining the emblematic character of the public building, promoting socialization, and becoming a center for the purchase of reference of local products in the city, and carry out this management with excellence.

Dividing the consolidated ISV by the amount of business volume (Lazkano et al., 2019), a ratio of social return on income of 2.31 is obtained. In other words, for every € 1 that the entity produces, it generates a value for society of € 2.31. Another ratio that could be interesting to monitor can be calculated by relating the figures obtained with the Cooperative's budget (Ayuso et al., 2020a). Specifically, dividing the consolidated ISV by the budget, a ratio of social performance of the Cooperative is obtained and amounts 2.08. In other words, the entity manages to generate a social value of € 2.08 for each € 1 of expenditure.

Figure 2 also shows the social value generated by the Cooperative for each stakeholder. To facilitate the understanding of representation, groups with similar interests have been put together, such as the city council, institutions, sponsors, and other markets into “institutions and companies.” The group that presented difficulties in being interviewed (customers) was not included, as no specific value variable was derived for it. In relation to the distribution of SSV to stakeholders, it is observed that the group that receives the highest value is that of partners, followed by personnel and institutions and companies. Likewise, ratios could be obtained for specific stakeholders, for example partners. Dividing the SSV received by the members of the Cooperative between their fees to the entity, a ratio of 6.72 is obtained. In other words, members receive a value of € 6.72 for every € 1 they contribute to the Cooperative.

## Discussions and Conclusions

### Specific Study Achievements

This research adds to the empirical literature focused on evaluation and orientation to social sustainability of Social Economy entities (Bull, 2007; Rotheroe and Richards, 2007; Bagnoli and Megali, 2011; Retolaza et al., 2014, 2015; Arena et al., 2015; Bassi and Vincenti, 2015; Etxezarreta et al., 2018; Lazkano et al., 2019; Ayuso et al., 2020a; Guzmán-Pérez et al., 2020; Román-Cervantes et al., 2020; Ruiz-Roqueñi, 2020). Specifically, this study focuses on the monitoring of social sustainability from the perspective of the measurement of social value, of urban food markets (Crespi-Vallbona et al., 2017; Alsadaty et al., 2020) self-managed through a cooperative associative formula. The ISV model (Retolaza et al., 2014, 2016) is used to derive the system of social sustainability indicators, and it is applied to a case study, the MNSA Cooperative, located on the island of Tenerife.

The measurement of the social value generated by the Cooperative and perceived by its stakeholders has made it possible to obtain a list of quantitative indicators referring to the organization's outputs valued by the stakeholders and reformulated in monetary units using financial approximations. The proposed metrics quantify different dimensions of the value perceived by the interest groups. These value variables can be regrouped into three categories, depending on whether they are considered to reflect the socio-economic functions of the supply markets, the behavioral traits of the SE or the practices of management and governance of the entity.

First, the variables “historical heritage,” “social relations,” “facilitates healthy shopping,” and “local consumption” include cultural and social aspects derived from the economic activity analyzed. These are manifestations of the sociocultural value promoted by urban food markets, identified from a theoretical point of view by the specialized literature on this topic. There are the historical and artistic importance of the buildings in which the marketing activity takes place and its potential to become a tourist attraction (Crespi-Vallbona and Domínguez-Pérez, 2016; Crespi-Vallbona et al., 2017), being public spaces for socialization and cultural interaction (Hiebert et al., 2014; Crespi-Vallbona and Dimitrovski, 2017; Alsadaty et al., 2020), the health benefits derived from the consumption of fresh products sold at their stalls (Rebollo and Casares, 2006; Costa et al., 2015) and the social and environmental utility of the locally-based urban food system that they promote (Pothukuchi and Kaufman, 1999; Casares, 2003; Connelly et al., 2011). These are aspects recognized as value by stakeholders and result from their experience of interaction with the cooperative.

Second, the dimensions “support services,” “way of life,” and “cooperation” are a direct reflection of two of the guiding principles of the Social Economy, the social purpose and solidarity or cooperation with the community (Mozas and Bernal, 2006; Chaves and Monzón, 2019). Specifically, “support services” implies recognition by the partners of the benefits that the mission of the entity brings them. In addition to the utility of the Cooperative for responding in a satisfactory way to the needs and problems of its members (Chaves and Monzón, 2018), the entity favors the integration of groups with fewer socioeconomic opportunities. This implies the foster of social cohesion that theoretically it is attributed to Social Economy entities (Bouchard, 2009; Chaves and Monzón, 2012). In this regard, it has been identified that members perceive the Cooperative as an instrument of economic support, enabling them to generate income and maintain the family unit (“livelihood”). Likewise, Third Sector entities and the local community value the entity's proactive support to the social mission that characterizes them or to the social projects they develop (“collaboration”). On the other hand, the subdimension “promotion of sectoral networks” of the variable “social relations” can be seen as a manifestation of the relational assets that emanate from the principle of cooperation inherent to these entities, and which would act as a catalyst for local and regional sustainability (Kim and Lim, 2017).

A final category of variables is made up of “customer attraction,” “leadership,” and “job satisfaction,” expressions of social value that come from the governance practices of the management team, and that *a priori* are not unique to the entities of the Social Economy. Although a lack of management capacity is a frequent problem among the Social Economy entities (Tomás-Carpi, 2008a), the identification of excellence in management as a dimension of the social value perceived shows the professionalization and ability of its management to face changes in commercial distribution and purchasing trends in Spanish cities (Casares and Rebollo, 1997), effectively providing a public service (Tomás-Carpi, 2008a).

The value variables relating to the functions of the urban food markets obtained in this study can be compared with the categories and fields evaluated by Crespi-Vallbona et al. (2017), taking into account differences between the two studies in terms of study design of the indicator system, as well as the methods used to measure sustainability. Thus, there is a coincidence between the variable “historical heritage” and the category “heritage under cultural protection,” and similarities between “local consumption” and “food market protection and integration in the city environment.” Analogies are also perceived in the indicators used to measure the variable “local consumption” and the subdimension “tourist attraction” of this study, with those of “air pollution” and “tourist volume demand” in Crespi-Vallbona et al. (2017), respectively. Failure to interview customers of the urban food markets managed by the Tenerife cooperative may explain the absence of more analogies in the results between the two works.

The phenomenological approach in the identification of the value variables and the fuzziness of the fair value do not allow a direct comparison of the results obtained between organizations, unless they have very similar characteristics or are from the same activity sector (Ayuso et al., 2020b; Freeman et al., 2020). However, the results of this study can be connected with those obtained by academic works that have applied the ISV model to other Social Economy entities, to determine possible parallels between the dimensions of perceived social value, or between the indicators and the proxies used.

The usefulness for the members of the MNSA Cooperative included in “support services” is also identified in the fishers' guild in Guzmán-Pérez et al. (2020), and can be considered analogous to the “cooperative return” of the consumer cooperative in Lazkano et al. (2019). The outputs and proxies used for the measurement in this work are the same as in the case of the guild, with certain differences with the consumer cooperative Lazkano et al. (2020) have internal data of the monetary value of discounts to members.

Secondly, the variable “collaboration” is similar to “volunteer activities” identified by Ayuso et al. (2020a) in a Special Employment Center, “participation of volunteers” in labor insertion enterprise in Retolaza et al. (2015), and “social response” in the Agrarian Transformation Society in Román-Cervantes et al. (2020). The indicators and proxies used are the same as those used by the rest of the jobs: the monetary value of the resources provided.

In addition, the variable “customer attraction” is similar to “brand strength” in Guzmán-Pérez et al. (2020) and “reputation and market expansion” in Román-Cervantes et al. (2020). The monetary quantification of the perceived social value in the MNSA Cooperative has been carried out using the same type of indicators and proxies.

The value variable “job satisfaction” is also identified in Guzmán-Pérez et al. (2020), estimating that it includes a social utility analogous to those of the set of value dimensions perceived by workers in Lazkano et al. (2020) and in Román-Cervantes et al. (2020). The approach to assessing this variable in the case here differs with respect to those studies, having chosen to approximate it through the monetary estimate of workers' surplus instead of using data on the number of partners or workers and using proxies referring to the Public Administration or commercial companies (Lazkano et al., 2019; Román-Cervantes et al., 2020).

A fifth similarity is observed between the “heritage” dimension of this study and that of “cultural promotion” of the Catalan Public Universities in Ayuso et al. (2020b), although there are differences in its measurement. “Heritage” has been quantified using data from competitive grants from the Public Administration, regional and national, while Ayuso et al. (2020b) have chosen not to express “cultural promotion” in monetary terms.

Finally, the value that reflects “local consumption” can be considered analogous to that of “reduction of CO_2_ emissions by recycling textile waste” in Retolaza et al. (2015), that of “promotion of social and environmental improvements” in Ayuso (2020), and to certain dimensions of “sustainability” in Román-Cervantes et al. (2020). However, the measurement perspective in this work has been different. Following Ayuso (2020), the value for people of the services provided by ecosystems to society has been quantified in monetary terms. For this, the social cost of carbon has been used as a proxy for estimating the savings in the carbon footprint derived from the transport of fruits and vegetables sold in the market stalls.

A result not related to the objectives of this study has been obtained, referring to the positioning of the different ecosystems of social value. Specifically, an SSV (non-market value) has been quantified for the MNSA Cooperative higher than the SEV (market value). This relative importance of the typologies of social value is similar to that obtained for the fishers' guild in Guzmán-Pérez et al. (2020) and for the grouping of agri-food cooperatives in Ruiz-Roqueñi (2020), although inverse to that of the consumer cooperative in Lazkano et al. (2020) and that of the social initiative cooperative in Etxezarreta et al. (2018). In order to explain the parallels and discrepancies between these cases, it would be necessary to take into account, in addition to the specific legal formulas, aspects such as the size of the entity, its age or the very dynamism of the market activity carried out. Ultimately, it would be necessary to extend the analysis to other organizations in this family of the Social Economy in order to determine whether there is an underlying pattern regarding the importance of the different categories of social value that they generate.

### Implications for Research and for the Industry

This study contributes to the scientific literature on measuring the social sustainability of Social Economy entities from the perspective of determining the social value generated. Its main contribution is derived from having focused the analysis on a service cooperative of an urban food market located in an island tourist destination, considered an ultra-peripheral region of the European Union, using the ISV system. The conjunction of these aspects has implications for the literature on the sustainability of urban supply markets and their measurement, Social Economy entities and the ISV model.

This work expands the research topic on the sustainability metrics for urban supply markets. It adds to the only existing system of indicators for urban supply markets, proposed by Crespi-Vallbona et al. (2017) following a top-down approach. The bottom-up orientation followed in this study, furthermore, has allowed to verify that the elements identified from a theoretical point of view on the potentiality of urban food markets to facilitate progress toward sustainable development (Casares, 2003; Costa et al., 2015; Crespi-Vallbona et al., 2017; Alsadaty et al., 2020) are perceived as value by stakeholders. This study also expands the existing evidence about the contribution of urban food markets to sustainability (Crespi-Vallbona et al., 2017; Alsadaty et al., 2020), through a case study for a market located in a geographic territory for which evidence was not available.

This research also supports theoretical works that identify Social Economy entities as levers of sustainable development (Connelly et al., 2011; Chaves and Monzón, 2012; Rahdari et al., 2016; Kim and Lim, 2017). The results obtained shows recognition as a source of value for stakeholders of the guiding principles of social aim and solidarity (Mozas and Bernal, 2006; Chaves and Monzón, 2018), as well as of the relational assets promoted by the principle of cooperation (Kim and Lim, 2017).

The results obtained contribute in two aspects to the progress of the ISV model toward the desired normalization (Retolaza et al., 2016; Ayuso et al., 2020b; Freeman et al., 2020). They allow advance in the identification of the interests that are common to the Social Economy (Lazkano et al., 2019; Guzmán-Pérez et al., 2020). They also reinforce the understanding of those interests that are shared by different forms of the Third Sector located in different territories of Spain. Likewise, this work has broadened the range of metrics for the quantification of certain dimensions of perceived social value. This last aspect, however, introduces greater variability in the system indicators.

The results obtained have three main implications for professionals in the sector. First, they provide relevant indicators of social sustainability (Aguado et al., 2015; Ayuso et al., 2020a) that can be integrated into management for an effective orientation of the strategy toward sustainability (Echanove, 2020; Freeman et al., 2020). Second, the financial expression of the social sustainability indicators favors the usefulness of these metrics as investment management and decision-making tools, since monetary measurement facilitates the integration and comparability of the indicators obtained with conventional financial and economic indicators (Aguado et al., 2015; Freeman et al., 2020). Finally, this measurement in monetary terms also facilitates communication and understanding by all stakeholders of the entity's non-financial information.

### Limitations and Future Research

This study has essentially three limitations, which are opportunities for the development of future research. The first two limitations are derived from the health context in which the analysis was carried out, the pandemic of COVID-19. Specifically, the interviews that were conducted by telephone or in small, enclosed spaces had a shorter average duration. This aspect could have had an impact on the degree of involvement of these interlocutors when expressing openly the perceived value in their relationship with the Cooperative. In the following process of analysis of the ISV of the entity that is opened, it would be convenient to carry out a greater number of face-to-face interviews with the groups that have been interviewed by telephone or in small spaces -workers, local entities, suppliers, city council, and institutions.

A second limitation of this analysis is that it does not include the interests of the group “customers.” The difficulties that arose in interviewing this group have meant that, in the analysis of the social value of the MNSA Cooperative, the specific dimensions of the social value that “customers” perceive have not been considered. Personal dialogue and the identification of possible specific value variables of this group would be necessary in subsequent analytical processes of the Cooperative's ISV. In this dialogue that is to be opened, it would also be necessary to have interlocutors who were local consumers and tourists, in order to analyze if there are differences in their interests and in their shopping experience in the MNSA positions.

A third limitation of this work derives from the phenomenological approach on which the ISV model is based. The results obtained could not be applied directly to another similar entity. For this, it would be necessary to have a set of cases, organizations of a similar nature and sector of activity, which would make it possible to specify shared interests and agree on outputs linked to the identified value variables. For their part, the proxies proposed for monetary quantification would have to take into account the different spatial-temporal realities in which the outputs were generated, although intervals of proxy values could be established that contemplate this diversity.

In any case, it would be necessary to analyze whether certain characteristics of the city in which this Market is located could limit the extrapolation of some of the value variables obtained here and their quantification to other supply markets in Spain. Specifically, Santa Cruz de Tenerife is located in an archipelago considered an outermost region of the EU. This feature could enhance the perception of the consumption of local products as a source of value (Barlagne et al., 2015), and its quantification is also likely to be higher in the case of the MNSA—due to the savings in carbon footprint derived from the transport of products from abroad.

A second aspect is related to the size and type of city in which the MNSA is located, a medium-size provincial capital. These features could affect the characterization of the dimension of perceived social value referring to the socialization that is promoted in the Market spaces (Tonkiss, 2005). Specifically, some of the attributes that define the variable “social relations” are those of closeness, cordiality or the promotion of values, traits that could differ from those that characterize socialization in the food markets of large cities such as Madrid or Barcelona. Consequently, it would be necessary to review the convenience of using the proxies used here for its quantification—annual membership fees for a social club in the city.

A final element to take into account when extrapolating to food markets in other cities is the importance of the city as a preferred destination for gastronomic tourists. In this case, Santa Cruz de Tenerife is mainly a destination for general tourists. Although these travelers include visits to gastronomic markets among their leisure activities, they do so to a lesser extent and their perceptions and interests are different from those of gastronomic tourists (Crespi-Vallobona, Dimitrovski and Crespi-Vallbona, 2017). This aspect should be considered in the extrapolation of the value variables identified in future dialogues with MNSA clients. Likewise, this element has partially influenced the quantification of the “heritage” variable in this study, so it should be considered when applying it to food markets in other cities.

## Data Availability Statement

The original contributions presented in the study are included in the article/Supplementary Material, further inquiries can be directed to the corresponding author.

## Ethics Statement

Ethical review and approval was not required for the study on human participants in accordance with the local legislation and institutional requirements. Written informed consent for participation was not required for this study in accordance with the national legislation and the institutional requirements.

## Author Contributions

BG-P, MP-M, and JM-J organized the data extracted from the interviews and wrote the first draft of the manuscript. All authors wrote sections of the manuscript, contributed to conception and design of the study, manuscript revision, read, and approved the submitted version.

## Conflict of Interest

The authors declare that the research was conducted in the absence of any commercial or financial relationships that could be construed as a potential conflict of interest.

## Footnotes

^1^To estimate the number of MNSA clients who are tourists, it has been assumed that the main reason for the trip of those who visit the island of Tenerife is not gastronomic, and that tourists who stay overnight in the city visit the Market. Taking into account that 32.61% of general tourists include among their activities the visit to gastronomic markets (Dinamiza Asesores, 2017, 43), and that in 2019 226,221 travelers stayed in the city of Santa Cruz de Tenerife (Canarian Statistics Institute), it can be estimated that 73,770 tourists visited the Market. This figure represents 7% of the customers that the Market had in 2019, according to internal data from the Cooperative.
