# Supplementary material for: Social Value and Urban Sustainability in Food Markets
Source: Front Psychol. 2021 Jun 16;12:689390. doi: 10.3389/fpsyg.2021.689390 (PMC8241922; doi:10.3389/fpsyg.2021.689390)
Supplement: Supplementary file 1 [file Table_1.docx]

Table 5: Valuation of variables of value

| **Variable of value** | **Stakeholders that receive value** | **Indicator** | **Value of indicator** | **Source of Indicator** | **Proxy** | **Proxy range** | **Given value** | **Source of proxy** | **Monetary value** |
| --- | --- | --- | --- | --- | --- | --- | --- | --- | --- |
| **Customer attraction** | Collaborators, Suppliers | Number of suppliers and collaborators who value it | 113 | Cooperative | Price of a sponsored ad on Facebook 5 days, to reach an audience of 15,000 people | €200€ | €200 | Facebook | €22,600 |
| **Supporting services** | Partners | Number of partners who value it | 119 | Cooperative | Difference between the annual cost of advisory,cleaning and security services, and the anual membership fee to the Cooperative | €1,911.39 - €4,810.95 | €3,361.17 | Consultation of prices of companies of advisory services, cleaning and security of the municipality.Cooperative | €399,979.23 |
| **Way of life** | Partners | Number of partners with fewer socioeconomic opportunities | 59 | Cooperative | Difference between the median annual salary inthe province and the Minimum Living Income | €13,762.41 | €13,762.41 | Instituto Nacional de Estadística. Salary Structure Survey(Canary Islands).  Royal Decree-Law 20/2020, of May 29, whichestablishes the Minimum Living Income | €676,112.27 |

| **Social relationships** | Partners, Workers, Town Hall, Other markets | Number of partners and workers who value it | 95 | Cooperative | Annual fee for a social club in the municipality | €660 - €928.08 | €794.04 | Consultation of prices of social clubs of the municipality | €75,433.80 |
| --- | --- | --- | --- | --- | --- | --- | --- | --- | --- |
|  |  | Number of groups that value it | 2 | Cooperative | Annual fee for a national profesional association | €108 - €576 | €342 | Price consultation of regional and national profesional associations | €684 |
| **Facilitates healthy shopping** | Partners, Workers | Number of partners and workers who value it | 36 | Cooperative | Annual amount of weekly home transport of abox of fresh fruit and vegetables | €160 | €160 | Consultation of prices of island companies that deliver fresh, organic fruit and vegetables | €5,760 |

| **Variable**  **de valor** | **Stakeholder que reciben valor** | **Indicador** | **Valor indicador** | **Fuente indicador** | **Proxy** | **Rango proxy** | **Valor atribuido** | **Fuente proxy** | **Valor monetario** |
| --- | --- | --- | --- | --- | --- | --- | --- | --- | --- |

| **Work satisfaction** | Workers | Worker surplus | €204,480.57 | Workers | - | | - | - | - | €204,480.57 |
| --- | --- | --- | --- | --- | --- | --- | --- | --- | --- | --- |
| **Historical heritage** | Partners, Suppliers,Collaborators, Town Hall, Institutions | Amount of competitive municipal grant  collection | €10,000 | Subsidies for the maintenance, restoration and/or conservation of properties of cultural value during the 2020 financial year—Cabildo de Tenerife. Official Gazette of the Province of Santa Cruz de Tenerife no. 87 20/07/2020 | | - | - | - | - | €10,000 |
|  | Partners, Collaborators, Town Hall | Number of tourist visits ^(1)^ | 73,770 | Dinamiza Asesores (2017).  Instituto Canario de Estadística. | | Entrance fee to the municipal museum | €7 | €7 | Price consultation of the Museum Tenerife Espacio de las Artes (TEA) | €516,390 |
|  | Partners, Institutions | Competitive national grant collection amount | €11,071.43 - €21,130.48 | Aid for projects for the conservation, protection and dissemination of properties declared World Heritage. 2019. Aid granted to dissemination projects. Resolution 06/06/2019. Undersecretary Ministry of Culture and Sports | |  |  | €16,100.91 | Aid for projects for the conservation, protection, and dissemination of properties declared World Heritage.2019. Aid granted to dissemination projects. Resolution06/06/2019. Undersecretary Ministry of Culture and Sports | €16,100.91 |

| **Variable**  **de valor** | **Stakeholder que reciben valor** | **Indicador** | **Valor indicador** | **Fuente indicador** | **Proxy** | **Rango proxy** | **Valor atribuido** | **Fuente proxy** | **Valor monetario** |
| --- | --- | --- | --- | --- | --- | --- | --- | --- | --- |

| ***Leadership*** |  | Producer surplus | €78,471.07 | Proveedores | - | - | - | - | €78,471.07 |
| --- | --- | --- | --- | --- | --- | --- | --- | --- | --- |
|  |  | Number of individuals who value it | 19 | Price consultation of ISO 9001 certified companies | Cost of certification in ISO 9001 | €1,400 - €3,000 | €1,500 | Price consultation of ISO 9001 certified companies | €28,500 |
| ***Collaboration*** | Local organizations | Amount of donated resources | €145,800 | Cooperative | - | €145,800 | €145,800 | Cooperative | €145,800€ |
|  |  | Amount of donated resources | €145,800 |  | Commercial credit interest rate | 2.346 % | 2.346 % | Maximum APR of the ICO Commercial Credit line effective from 11/30/2020 to 12/06/2020 | €3,420.47 |
| ***Local consumption*** | Institutions | Savings in the carbon footprint of transporting fruit and vegetables purchased by customers ^(4), (5), (6)^ | 698.15 Ton CO_2_ |  | Social cost of carbon | 40.18 €/Ton CO_2_ – 158.04 €/Ton CO_2_ | 99.11 €/Ton CO_2_ | García Pérez (2019)  Exchange rate €/$ Banco de España (2019) | €69,191.85 |

(1) Estimated value, taking into account that 32.67% of general tourists, or non-gastronomic tourists, visit gastronomic markets (Dinamiza Asesores, 2017: 42), and that the number of tourists staying in Santa Cruz de Tenerife in 2019 amounted to 226,221 (Canarian Statistics Institute).

(3) Dissemination projects: “III International Congress. Mudéjar world heritage “The Mudejar trades: past, present and future” (Tobed Town Hall), and“ Study and dissemination of Pastoralism in the Pyrenees-Monte Perdido IV ”(Sobrarbe region).

(4) According to the Ministry of Agriculture, Livestock and Fisheries (2020), in the Canary Islands the average consumption per person in 2018 of fruits and vegetables was 87.2 kg and 50.23 kg, respectively.

(5) Carbon footprint of transport estimated with the calculator available at Alimentoskilometricos.org.

(6) The average number of clients of the Nuestra Señora de África Market is 20,384 per week, according to data from the MNSA Cooperative.

Source: Authors’ elaboration

Table 6: Socio Economic Direct Value

| **Socio Economic Direct Value** | | **€575,273.74** |
| --- | --- | --- |
| **Added and distributed value** | | **€484,195.03** |
|  | *Net salaries* | €342,704.69 |
|  | *Dividends* | 0.00 |
|  | *Social Security* | €127,106.11 |
|  | *IRPF* | €13,285.75 |
|  | *Other taxes* | €18,147.68 |
|  | *IGIC* | €-18,183.94 |
|  | *Taxes over benefits* | €0.00 |
|  | *Financial suppliers* | €1,134.74 |
| **Added and retained value** | | **€21,078.71** |
|  | *Reserves* | €0.00 |
|  | *Non distributed results* | €21,078.71 |
|  | *Amortizations* | €0.00 |

*Source: Authors’ elaboration.*

Table 7: Repercusion indexes in suppliers

| **Descripción** | **Operational suppliers** | **Investment suppliers** |
| --- | --- | --- |
| *Suppliers turnover* | 100.00% | 100.00% |
| *Personnel expenditures* | 27.43% | 49.04% |
| *Added value* | 35.90% | 54.85% |
| *Yearly results* | 4.19% | 3.64% |
| *Taxes* | 1.35% | 0.35% |

*Source: Authors’ elaboration.*

Table 8: Indirect Socio-Economic Value.

|  | **Operational suppliers** | | **Investment suppliers** |
| --- | --- | --- | --- |
| **Indirect Socio-Economic Value** | **€129,451.73** | **€108,999.46** | |
| *Net salaries* | €51,085.31 | €49,674.09 | |
| *Social Security* | €34,391.49 | €33,441.44 | |
| *IRPF* | €7,473.18 | €7,266.73 | |
| *IGIC* | €17,712.66 | €11,248.12 | |
| *Taxes* | €4,588.63 | €651.70 | |
| *Yearly results* | €14,200.46 | €6,717.38 | |

*Source: Authors’ elaboration.*

Table 9: Social Value Ecosystems

| **Social Value Ecosystems** | **Valor monetario** |
| --- | --- |
| **Valor Social de Mercado** | **€743,724.93** |
| Direct Socio-Economic Value | €505,273.74 |
| Indirect Socio-Economic Value | €238,451.19 |
| *- Operational Suppliers* | *€129,451.73* |
| *- Investment Suppliers* | *€108,999.46* |
| **Specific Social Value** | **€2,252,.924.07** |
| **Integrated Social Value** | **€2,996,649.00** |

*Source: Authors’ elaboration.*

**References:**

Dinamiza Asesores (2017). II Estudio de la Demanda de Turismo Gastronómico en España. Madrid: Dinamiza Asesores.

Ministerio de Agricultura, Ganadería y Pesca (2020). Informe del Consumo de Alimentación en España 2019. Madrid: Ministerio de Agricultura, Ganadería y Pesca.
